# From Diet to Scar: Novel Mendelian Randomization and Mediation Analyses Linking Dietary Habits, Gut Microbiota, and Hypertrophic Scarring

**DOI:** 10.1002/fsn3.70292

**Published:** 2025-05-28

**Authors:** Qiong Liu, Xiaofang Liu, Mengge Gao, Bo Yang, Miaoqing Luo, Biying Yang, Guojun Liang

**Affiliations:** ^1^ Department of Clinical Nutrition Huadu District People's Hospital of Guangzhou Guangzhou Guangdong China; ^2^ Surgical Department First Affiliated Hospital of Guangzhou Medical University Guangzhou Guangdong China

**Keywords:** co‐localization analysis, dietary habits, gut microbiota, hypertrophic scarring, mendelian randomization

## Abstract

Hypertrophic scarring (HTS) is a pathological skin condition characterized by excessive collagen deposition during wound healing. Emerging evidence suggests that dietary habits and gut microbiota composition may influence HTS risk via systemic inflammatory and metabolic pathways. However, the causal relationships between these factors remain poorly understood. Mendelian randomization (MR) analysis was conducted to investigate the causal relationships between dietary habits, gut microbiota composition, and HTS risk. Additional analyses included mediation analysis to explore potential intermediary effects of gut microbiota and co‐localization analysis to assess shared genetic loci between exposures and HTS. MR analysis identified significant associations between HTS and six dietary preferences, with caffeinated/sweet liking and jam liking increasing HTS risk, while crisps, curry, oranges, and strong flavor liking were protective. For gut microbiota, *Eubacterium coprostanoligenes* , Collinsella, and Coprococcus1 showed protective effects, whereas Adlercreutzia was positively associated with HTS risk. Mediation analysis did not support gut microbiota as a significant mediator between dietary habits and HTS, and co‐localization analysis indicated distinct genetic determinants for these traits. The study highlights the independent roles of dietary habits and gut microbiota in influencing HTS risk, suggesting potential dietary and microbial‐targeted interventions for scar prevention. Further research in diverse populations is needed to validate these findings and explore their clinical applications.

## Graphical abstract

Our Mendelian randomization study demonstrated that specific dietary preferences and gut microbial taxa independently influence the risk of hypertrophic scarring (HTS). Dietary habits such as reduced intake of refined sugars and increased consumption of antioxidant‐rich foods were protective. Gut microbial composition also contributed to HTS susceptibility through distinct, non‐overlapping biological pathways.

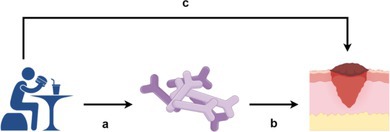

## 1 Introduction

Hypertrophic scarring (HTS) represents a pathological response to dermal injury, characterized by excessive collagen deposition that leads to raised, erythematous, and often pruritic lesions that remain confined within the boundaries of the original wound (Hu et al. 2020). These scars typically develop following dermal trauma such as burns, surgical incisions, or lacerations and, although they may exhibit spontaneous regression over time, frequently persist to cause substantial physical discomfort, functional impairment, and psychological distress. While HTS is distinct from keloids in that they do not extend beyond the wound margins, their localized nature does not mitigate the complexities associated with their development, management, and treatment (Silva et al. 2015).

The formation of HTS is underpinned by a multifaceted and incompletely understood interplay of genetic, mechanical, and biochemical factors that disrupt the wound healing process, particularly during the proliferative and remodeling phases. Central to this pathological process is an aberrant activation of fibroblasts, resulting in an overproduction of extracellular matrix components, predominantly type I and III collagen. This process is further perpetuated by dysregulated signaling cascades, including overexpression of profibrotic mediators such as transforming growth factor‐beta (TGF‐β), which sustains chronic inflammation and promotes excessive tissue remodeling. Despite extensive research into these molecular and cellular mechanisms, substantial gaps remain in the understanding of the precise factors and pathways that dictate the development and progression of HTS, thereby limiting the efficacy and consistency of existing therapeutic interventions (Vatandoost et al. 2016; Carter et al. 2016).

While advancements in treatments such as silicone gel sheeting, intralesional corticosteroid injections, laser therapies, and surgical excision have provided clinicians with a broader arsenal of therapeutic options, these interventions often yield heterogeneous outcomes and are frequently accompanied by the risk of recurrence. Consequently, there is an urgent need to explore novel etiological and modulatory factors that may influence HTS formation and to develop innovative strategies for prevention and treatment. Emerging evidence suggests that dietary habits, which can influence the composition and diversity of the gut microbiota, may serve as key modulators of these systemic inflammatory and immune pathways (Ferrari et al. 2022; Pride et al. 2021). Diet is known to affect the gut microbiota's functionality, and alterations in microbial populations can lead to dysbiosis, a condition associated with chronic inflammation and fibrotic disorders. Specifically, certain dietary patterns have been implicated in exacerbating inflammatory responses, potentially contributing to conditions like HTS. For example, diets high in refined sugars or unhealthy fats can promote an inflammatory environment that may impair the normal healing process and enhance scar formation. Conversely, diets rich in fiber, antioxidants, and anti‐inflammatory compounds may help restore gut homeostasis and mitigate the risk of excessive scarring (Zhao et al. 2018; Al‐Obaide et al. 2017).

Although the potential for dietary patterns and gut microbiota to influence HTS is intriguing, the precise mechanisms linking these factors to scar formation are not yet well understood. Current evidence is largely associative, with studies suggesting that diet‐induced alterations in gut microbiota composition may impact wound healing through their effects on inflammatory and fibrotic pathways. As such, further research is needed to explore the causal relationships between diet, gut microbiota, and HTS formation (Karl et al. 2022). Understanding how these factors interact could reveal novel preventive strategies and therapeutic interventions, providing new avenues for reducing the burden of HTS and improving patient outcomes (Canale et al. 2021). By elucidating the connections between diet, gut microbiota, and scar formation, future studies may pave the way for more effective treatments that not only address the physical aspects of scarring but also enhance quality of life by reducing the psychological impact of hypertrophic scars.

The exploration of causal relationships between dietary habits, gut microbiota, and HTS is complex, as traditional observational studies are susceptible to confounding factors and reverse causation. While these studies can identify associations, they fail to definitively establish causality, especially when exposures like diet and gut microbiota are influenced by numerous external factors. Mendelian randomization (MR) addresses these limitations by using genetic variants as instrumental variables (Neil et al. 2018). These genetic variants are randomly assigned at conception, independent of confounders and not subject to reverse causality, allowing for more reliable causal inferences. MR uses genetic proxies associated with specific exposures, such as diet or gut microbiota, to estimate their causal effects on HTS development, providing a powerful method to explore the potential causal pathways between these factors.

The biological plausibility of this investigation is supported by the known roles of diet and gut microbiota in modulating immune and inflammatory pathways central to wound healing. Dysbiosis and inflammation have been linked to impaired tissue repair and fibrotic disorders, including HTS. However, direct evidence of causality remains scarce, with most studies being observational. MR provides a rigorous solution to this gap, enabling an investigation of the causal relationship while minimizing confounding biases (Qi et al. 2024). Additionally, MR can be paired with colocalization analysis, further strengthening the findings by ensuring the genetic associations between exposures and HTS are not confounded by pleiotropy.

The primary objectives of this study are to estimate the causal effects of dietary habits and gut microbiota on HTS risk using two‐sample MR. Specifically, this study aims to assess whether gut microbiota mediates the relationship between dietary habits and HTS, and to explore whether these causal pathways can inform new preventive or therapeutic approaches to HTS. Furthermore, the study will use colocalization analysis to confirm that the observed genetic associations between exposures and HTS are not driven by pleiotropic effects, thereby providing robust evidence for the causal relationships under investigation.

## 2 Materials and Methods

### 2.1 Study Design

**FIGURE 1:**
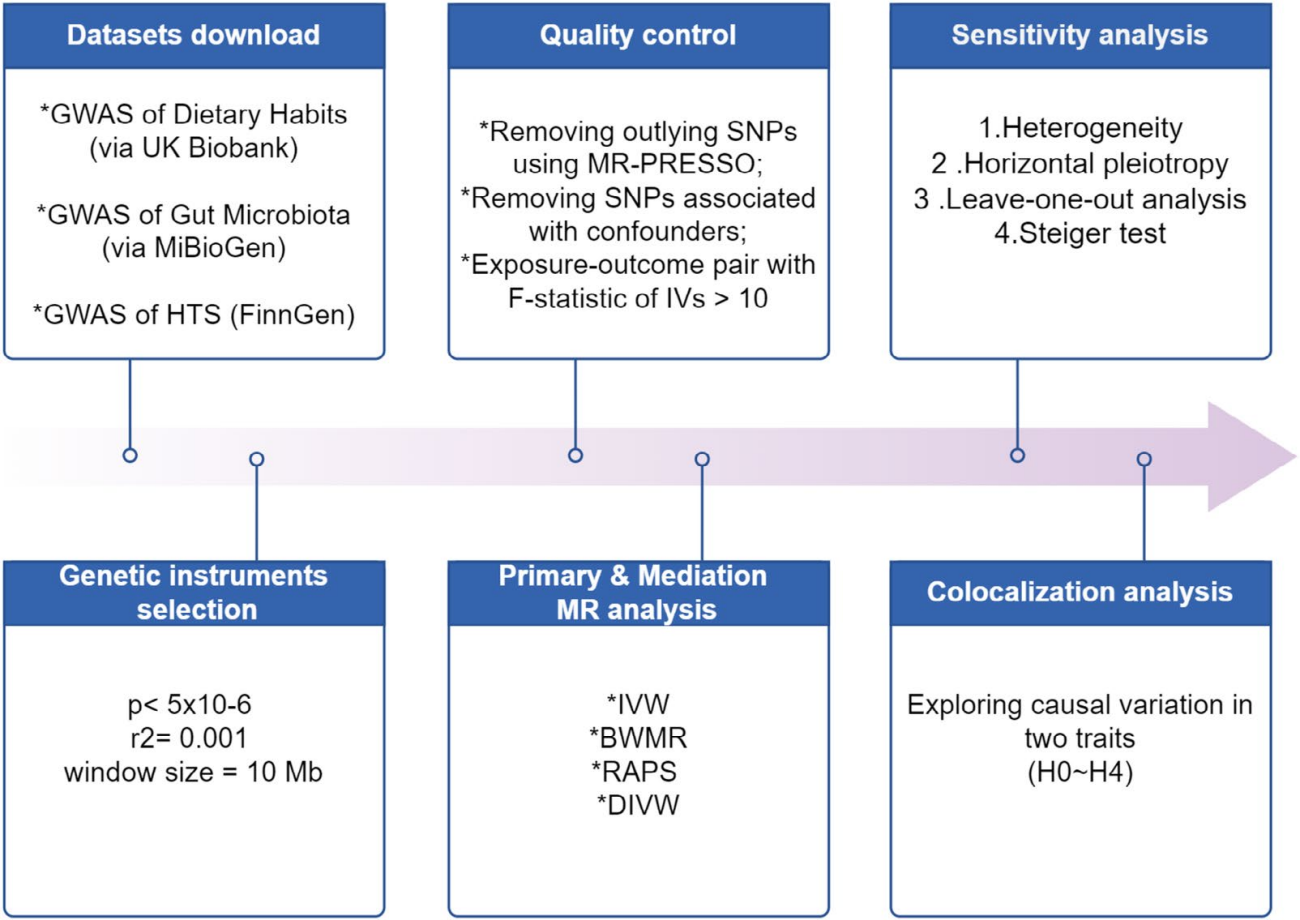
Overview of the research and design of the study. By *Figdraw*.

A comprehensive MR design is employed to investigate potential causal pathways between dietary habits, gut microbiota, and HTS. The research is structured in three key stages: an initial MR analysis to examine the direct causal effects of dietary habits and gut microbiota on HTS, followed by mediation analysis, and concluding with colocalization analysis (Figure 1).

The first stage focuses on assessing whether dietary habits and gut microbiota composition independently influence HTS risk. In this stage, dietary patterns and features of the gut microbiota are considered as exposures, with HTS serving as the outcome. The second stage examines whether gut microbiota mediates the relationship between dietary habits and HTS. In this step, dietary habits are treated as the primary exposure, gut microbiota as the potential mediator, and HTS as the outcome. This mediation analysis aims to clarify whether changes in gut microbiota composition could explain how dietary habits influence HTS formation, offering deeper insight into the biological mechanisms at play.

Finally, colocalization analysis will be applied to the significant MR results to explore whether shared genetic loci contribute to both gut microbiota composition and HTS development. This analysis will help identify whether the genetic basis for microbiota composition overlaps with that of HTS, offering a clearer understanding of the shared genetic factors that could explain the observed causal relationships. This approach ensures that identified associations are both causal and genetically supported, strengthening the reliability of the findings.

### 2.2 Instrumental Variables

Single nucleotide polymorphisms (SNPs) are selected as instrumental variables (IVs) to assess the causal relationship between dietary habits, gut microbiota, and HTS. To ensure robust and accurate findings, the chosen SNPs must exhibit strong associations with the exposures—dietary habits and gut microbiota—and meet specific criteria for inclusion in the MR analysis. SNPs associated with dietary habits and gut microbiota composition were identified using a significance threshold of *p* < 5 × 10^−6^ to ensure moderate statistical significance (Du et al. 2023). This threshold avoids excluding relevant instruments while ensuring an adequate number of SNPs for analysis. A stricter genome‐wide threshold (*p* < 5 × 10^−8^) would have reduced the number of SNPs too significantly, limiting the statistical power of the study.

To minimize bias from correlated genetic variants, linkage disequilibrium (LD) pruning was performed, excluding SNPs in high LD (*r* ^2^ > 0.001) within a 10,000 kb window (Gazal et al. 2017). This ensures the independence of the IVs, as correlated genetic variants could distort causal inference. The quality of the SNPs as instruments was further assessed using the F‐statistic, and SNPs with an F‐statistic below 10 were excluded due to the potential risk of weak instrument bias (Levin et al. 2020; Pierce et al. 2011).

Moreover, pleiotropy tests were conducted to ensure the validity of the IVs, excluding SNPs that might directly affect HTS or any confounders influencing both exposure and outcome. The LDlink database was used to identify and exclude SNPs associated with potential confounders (Machiela and Chanock 2015). For the analysis, the TwoSampleMR and MRInstruments R packages were employed, providing robust tools for selecting and validating genetic instruments. Through these rigorous SNP selection and validation protocols, the reliability and independence of the IVs were ensured, enhancing the credibility of the causal inferences (Yavorska and Burgess 2017; Ong and MacGregor 2019).

## 3 Data Sources

### 3.1 Genome‐Wide Association Study (GWAS) Data Sources for Dietary Habits

Summary‐level data on dietary preferences were obtained from a genome‐wide association study involving 161,625 participants of European ancestry in the UK Biobank. Food preferences were assessed using a standardized 9‐point scale across 139 food items, and genetic associations were estimated under rigorous quality control procedures (May‐Wilson et al. 2022). Participants were aged between 40 and 69 years, with phenotypic data such as age, BMI, and health history collected at baseline.

Individual‐level data on dietary quantity, clinical status, and probiotic use were not available for inclusion in this summary‐level analysis, as is typical of publicly shared GWAS datasets. This feature has been accounted for when evaluating the potential for residual confounding, especially for exposures such as age‐related microbiota variation.

### 3.2 Sources of GWAS Data on Gut Microbiota

Gut microbiota data were derived from the MiBioGen consortium, which conducted a meta‐analysis across 24 international cohorts, encompassing 18,340 participants, approximately 85% of whom were of European ancestry. Taxonomic profiling was based on 16S rRNA gene sequencing and annotated using the Ribosomal Database Project (RDP) classifier. Genetic loci regulating microbiota composition were identified through multivariate models assessing microbiome quantitative trait loci (mbQTL) and trait loci (mbTL) (Kurilshikov et al. 2021). As with the UK Biobank dietary dataset, individual‐level variables such as probiotic supplementation or comorbid conditions were not available in the summary statistics. Interpretations of microbial associations were thus made with consideration of these typical constraints.

### 3.3 GWAS Data Sources for HTS

The data on HTS for this study was sourced from the FinnGen R12 release (r12.finngen.fi), which includes comprehensive genomic and clinical data from participants in the Finnish biobank. The HTS cases in this dataset were identified using the ICD‐10 code L91.0, which covers hypertrophic scars and keloid scars. The dataset contains 2068 HTS cases and 465,673 controls, ensuring a robust sample size for genetic analysis. The data includes additional metadata, such as classification under the ICD hierarchy (Level 4), and was first used in the DF2 data freeze. The ICD‐10 parent code for HTS is L91, with exclusions for acne keloid (L73.0) and scar NOS (L90.5). Genetic analysis was conducted to identify variants associated with HTS, providing valuable insights into the genetic factors influencing this condition. All summary data used in this study were based on harmonized cohorts and passed genome‐wide quality control filters.

### 3.4 Assumptions

**FIGURE 2:**
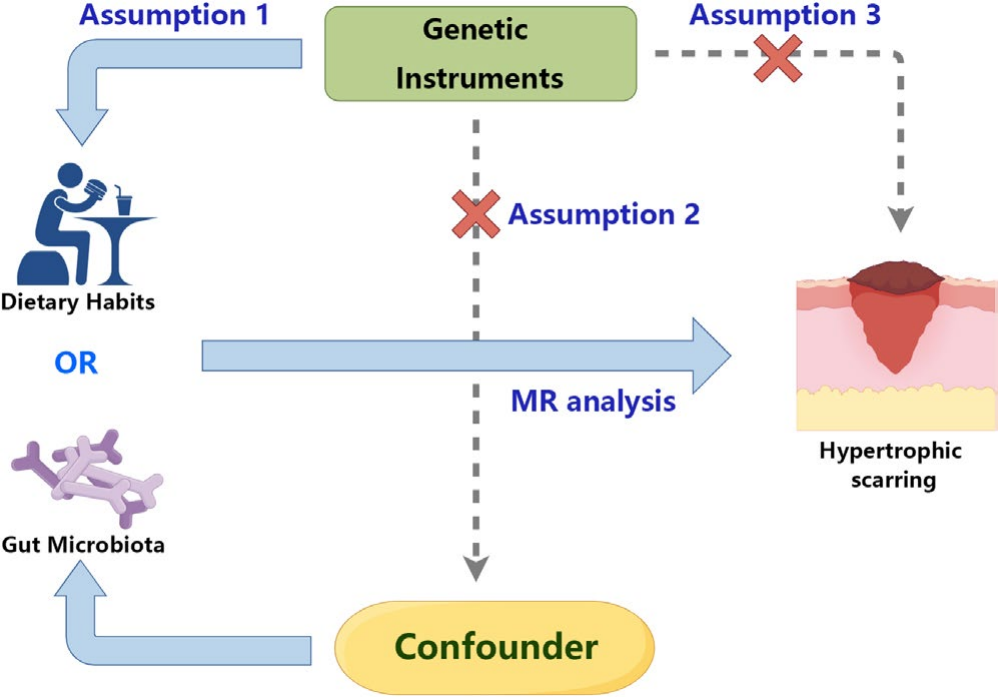
Schematic diagram of our Mendelian randomization assumptions.

In this study, several key assumptions underpin the MR framework used to explore the causal relationships between dietary habits, gut microbiota, and HTS. First, it is assumed that the genetic variants used as IVs are strongly associated with the exposures—dietary habits and gut microbiota composition—and that this association remains robust across the studied population (Meng et al. 2009). Second, the genetic instruments must influence the outcome, HTS, only through the exposure of interest, without confounding pathways, ensuring that there is no horizontal pleiotropy (de Bakker et al. 2006). This assumption is critical for maintaining the integrity of causal inference, as any pleiotropic effect would violate the exclusion restriction required for valid IV estimation. Third, the study assumes that the genetic variants used in the MR analysis are not affected by reverse causation, meaning that the genetic variants influencing dietary habits and gut microbiota composition precede the development of HTS (Lawlor et al. 2008). This temporal relationship is essential to avoid spurious associations arising from reverse causality. Finally, the analysis assumes that the samples used for the GWAS data on exposures and outcomes are independent, ensuring that the results from the two‐sample MR design remain valid. These assumptions are fundamental to the MR approach and the interpretation of causal relationships in the study, ensuring that the findings reflect the true biological effects rather than confounding or reverse causality (Figure 2).

## 4 Statistical Methods

### 4.1 Main Analysis

Several advanced MR methods are employed to enhance the robustness and accuracy of causal inferences regarding the relationships between dietary habits, gut microbiota, and HTS. The Inverse Variance Weighted (IVW) method serves as the standard approach, combining associations between genetic variants, exposures, and outcomes to estimate causal effects, assuming no horizontal pleiotropy (Burgess et al. 2015). However, to address potential bias from weak instruments, the Debiased Inverse Variance Weighted (DIVW) method is used, which adjusts for weak instrument bias and provides more reliable estimates, especially when the genetic instruments are not strong enough—a limitation that the traditional IVW method may overlook (Habibi et al. 2024). In addition, the Robust Adjusted Profile Score (RAPS) method is applied to account for pleiotropy, particularly systematic pleiotropy, by utilizing robust loss functions like Tukey or Huber, which minimize the influence of outliers and weak instruments (Yu, Chen, et al. 2023). Finally, the Bayesian Weighted Mendelian Randomization (BWMR) approach is incorporated, allowing for the inclusion of prior distributions within a Bayesian framework, which leads to more precise causal estimates and a better understanding of uncertainty in the analysis. These methods are superior to traditional MR techniques like MR‐Egger and weighted median, particularly in mitigating biases and improving statistical power, ensuring a comprehensive and reliable exploration of the causal pathways in the study (Zhao et al. 2020).

### 4.2 Mediation Analysis

**FIGURE 3:**
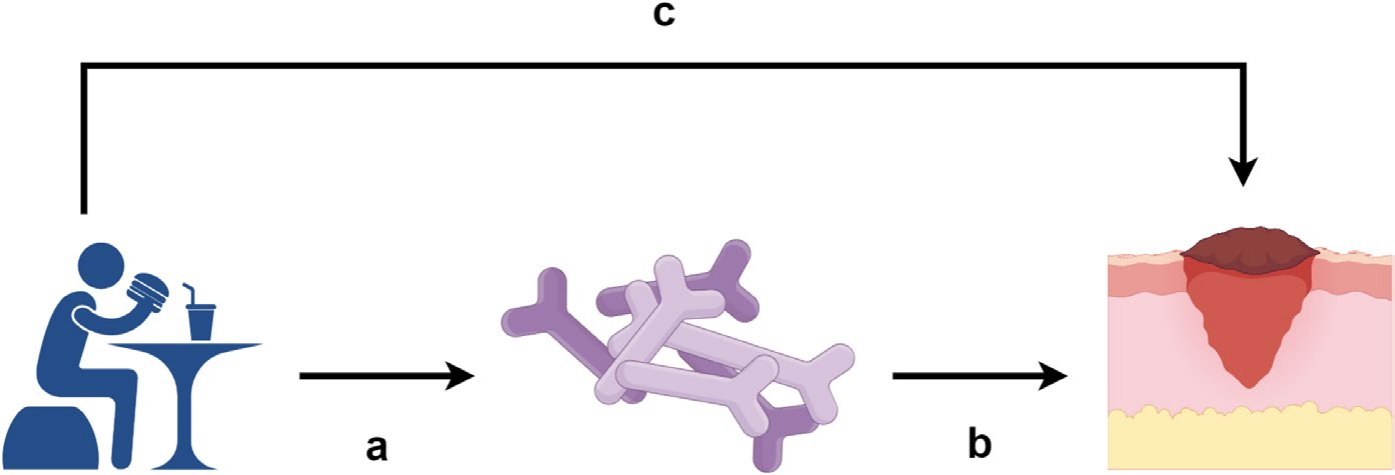
Conceptual framework for the mediation analysis of dietary habits, gut microbiota, and HTS.

A mediation analysis is performed to explore whether gut microbiota mediates the relationship between dietary habits and HTS. The mediation analysis is performed to assess whether gut microbiota mediates the relationship between dietary habits and HTS. The analysis follows a series of steps to estimate the indirect effect of dietary habits on HTS through gut microbiota. First, genetic variants associated with dietary habits are used as instrumental variables to assess the impact of dietary exposure on gut microbiota composition. Second, gut microbiota composition is treated as the mediator, and its influence on HTS is evaluated. The primary statistical model used is a causal mediation model, where the total effect of dietary habits on HTS is decomposed into direct and indirect effects. The indirect effect is quantified by estimating the portion of the effect that occurs through changes in gut microbiota composition. This approach allows for the identification of potential pathways by which dietary habits could influence HTS through gut microbiota (Figure 3). Sensitivity analyses are conducted to test the robustness of the findings, ensuring that the mediation model assumptions are met and that the results are not influenced by confounding or pleiotropy (Sanderson 2021).

### 4.3 Sensitivity Analyses and Additional Analyses

The sensitivity analyses conducted in this study aim to validate the robustness of the causal estimates and ensure that the results are not driven by confounding factors or biases. To evaluate directional pleiotropy, MR‐Egger regression is employed, with a particular focus on the intercept test (Bowden et al. 2015). A non‐zero intercept would indicate that some genetic instruments might affect the outcome through pathways unrelated to the primary exposure, potentially introducing bias. To further refine the results and adjust for pleiotropic outliers, the MR‐PRESSO method is applied (Verbanck et al. 2018). Additionally, a leave‐one‐out analysis is performed, sequentially excluding each genetic variant to ensure that no single SNP disproportionately influences the overall causal estimate. Cochran's Q statistic is also calculated to assess the heterogeneity of the genetic instruments, providing insight into the consistency of the instruments used in the analysis (Burgess et al. 2013). Finally, the Steiger test is employed to confirm that the orientation of the genetic variants is correct, ensuring that the associations between SNPs and exposures are appropriately aligned for valid causal inference (Lutz et al. 2022). These sensitivity analyses collectively strengthen the reliability of the findings.

### 4.4 Co‐Localization Analysis

In addition to the primary MR analysis, co‐localization analysis was conducted to examine whether the same genetic variants are involved in both the exposures—dietary habits and gut microbiota—and HTS. This method estimates the posterior probabilities for several competing hypotheses: H0 (neither trait is associated with the genetic variant), H1 (only dietary habits or gut microbiota is associated), H2 (only HTS is associated), H3 (both traits are associated, but through distinct causal variants at the same locus), and H4 (both traits are associated and share the same causal variant). The primary focus is on hypothesis H4, as it suggests that the genetic variants influencing both dietary habits or gut microbiota and HTS are the same. A high posterior probability for H4 would indicate that the same genetic variants contribute to both the exposures and HTS, providing strong evidence for a shared genetic basis between these factors (Giambartolomei et al. 2014). This shared genetic architecture would support the idea that the genetic variants driving dietary habits or gut microbiota composition also play a role in the development of HTS, offering insight into the genetic mechanisms underlying the condition.

All statistical analyses were performed using R‐Studio (version 4.4.1) with specialized packages tailored to meet the requirements of each analytical step. Mendelian randomization was conducted with the TwoSampleMR package, while pleiotropy was assessed using MR‐PRESSO. Bayesian Weighted Mendelian Randomization was implemented with the BWMR package, and MR‐RAPS was utilized for robust adjusted profile score analysis. Co‐localization analysis was carried out using the coloc package.

## 5 Results

### 5.1 Effect of Food Liking on HTS

**FIGURE 4:**
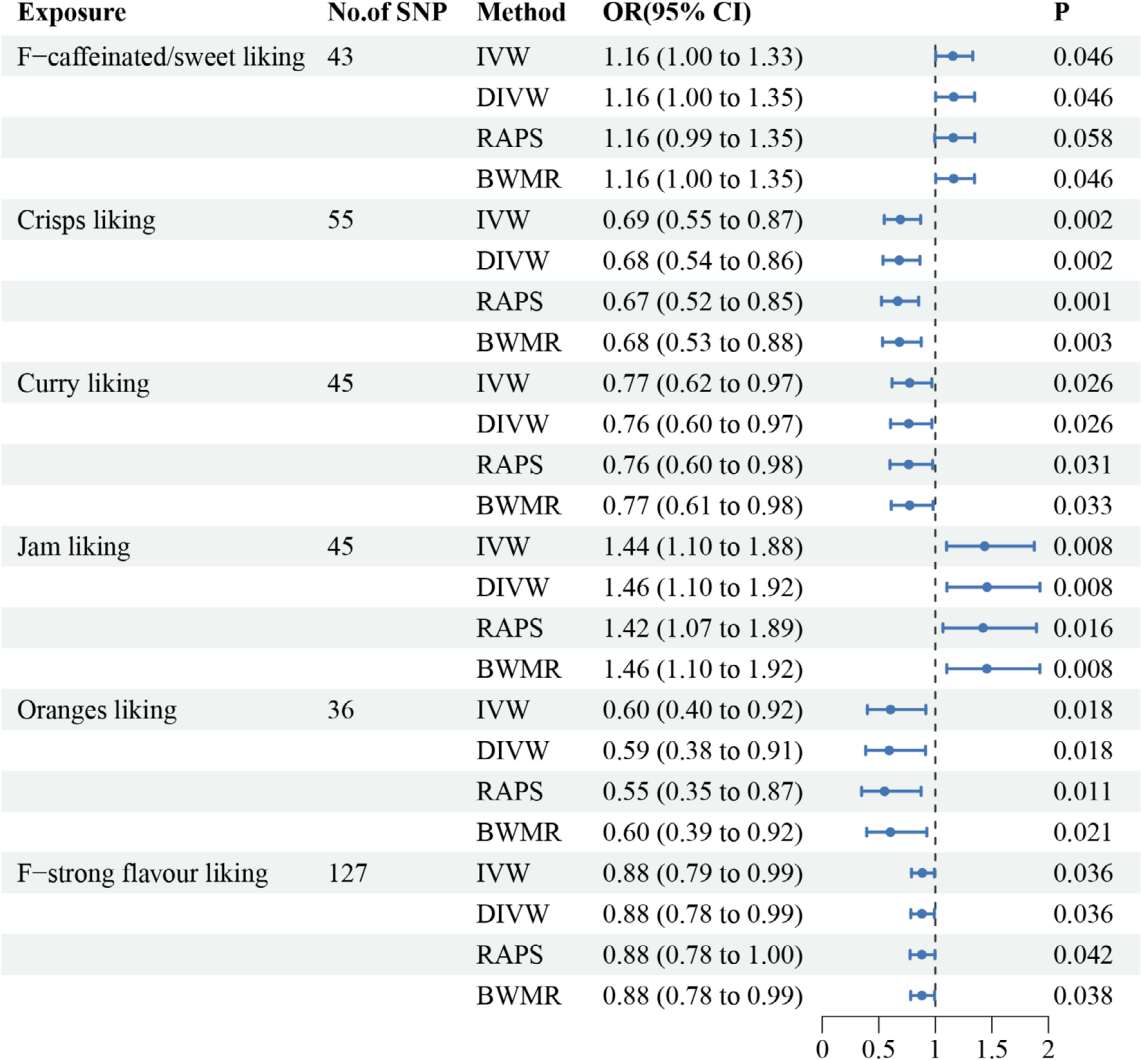
MR estimates for the associations between dietary habits and genetically predicted HTS. CI, confidence interval; OR, odds ratio.

**FIGURE 5:**
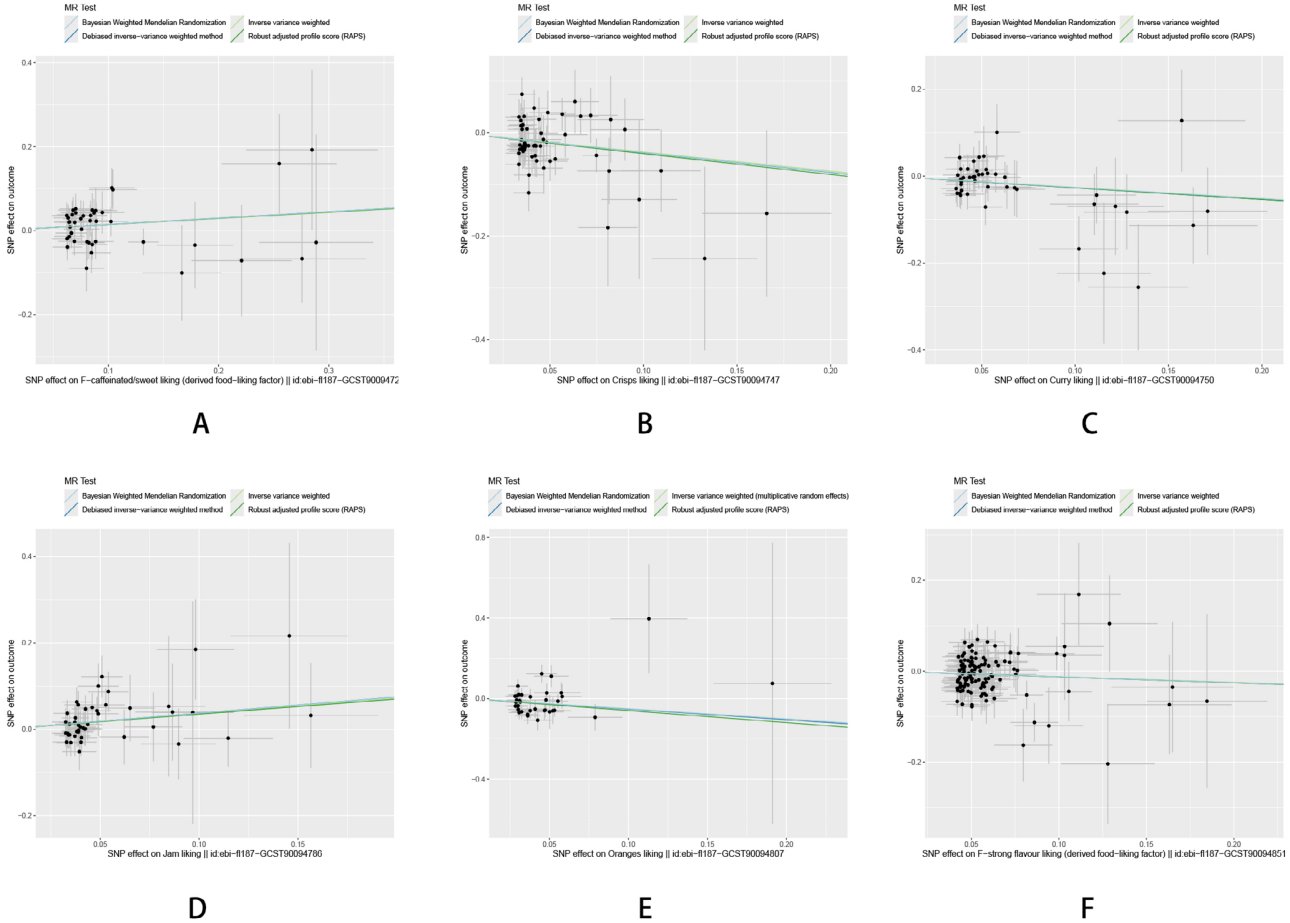
Scatter plots illustrating MR estimates for the associations between dietary habits and genetically predicted HTS. OR: odds ratio; CI: confidence interval. Panels (A–F) represent the associations for F‐caffeinated/sweet liking (A), crisps liking (B), curry liking (C), jam liking (D), oranges liking (E), and F‐strong flavor liking (F), depicting the SNP‐based exposure effects plotted against HTS risk.

The MR analysis assessed the causal relationship between various dietary preferences and the risk of HTS, identifying six significant associations. F‐caffeinated/sweet liking was positively associated with HTS (OR 1.16, 95% CI 1.00 to 1.33, *p* = 0.046). Crisps liking showed a protective effect, with a significant inverse association (OR 0.69, 95% CI 0.55 to 0.87, *p* = 0.002). Similarly, curry liking and jam liking were also significantly associated with HTS, with odds ratios of 0.77 (95% CI 0.62 to 0.97, *p* = 0.026) and 1.44 (95% CI 1.10 to 1.87, *p* = 0.008), respectively. Oranges liking and strong flavor liking showed weaker associations but were still statistically significant (OR 0.60, 95% CI 0.40 to 0.92, *p* = 0.018; OR 0.89, 95% CI 0.79 to 0.99, *p* = 0.036). The above results are illustrated in Figures 4 and 5 and Table S1.

Sensitivity analyses were conducted to evaluate the robustness of the MR findings and to identify potential violations of assumptions, including pleiotropy and heterogeneity. Across most exposures, no evidence of pleiotropy or heterogeneity was observed, supporting the reliability of the causal estimates. For oranges liking, however, heterogeneity was detected. The Cochran's Q statistic for the inverse variance weighted (IVW) method was 55.98 (degrees of freedom [df] = 35, *p* = 0.014), and for the MR‐Egger method, it was 55.40 (df = 34, *p* = 0.012). These findings suggest that the genetic instruments for oranges liking may exhibit some variability, which could influence the precision of the causal estimates for this exposure.

**FIGURE 6:**
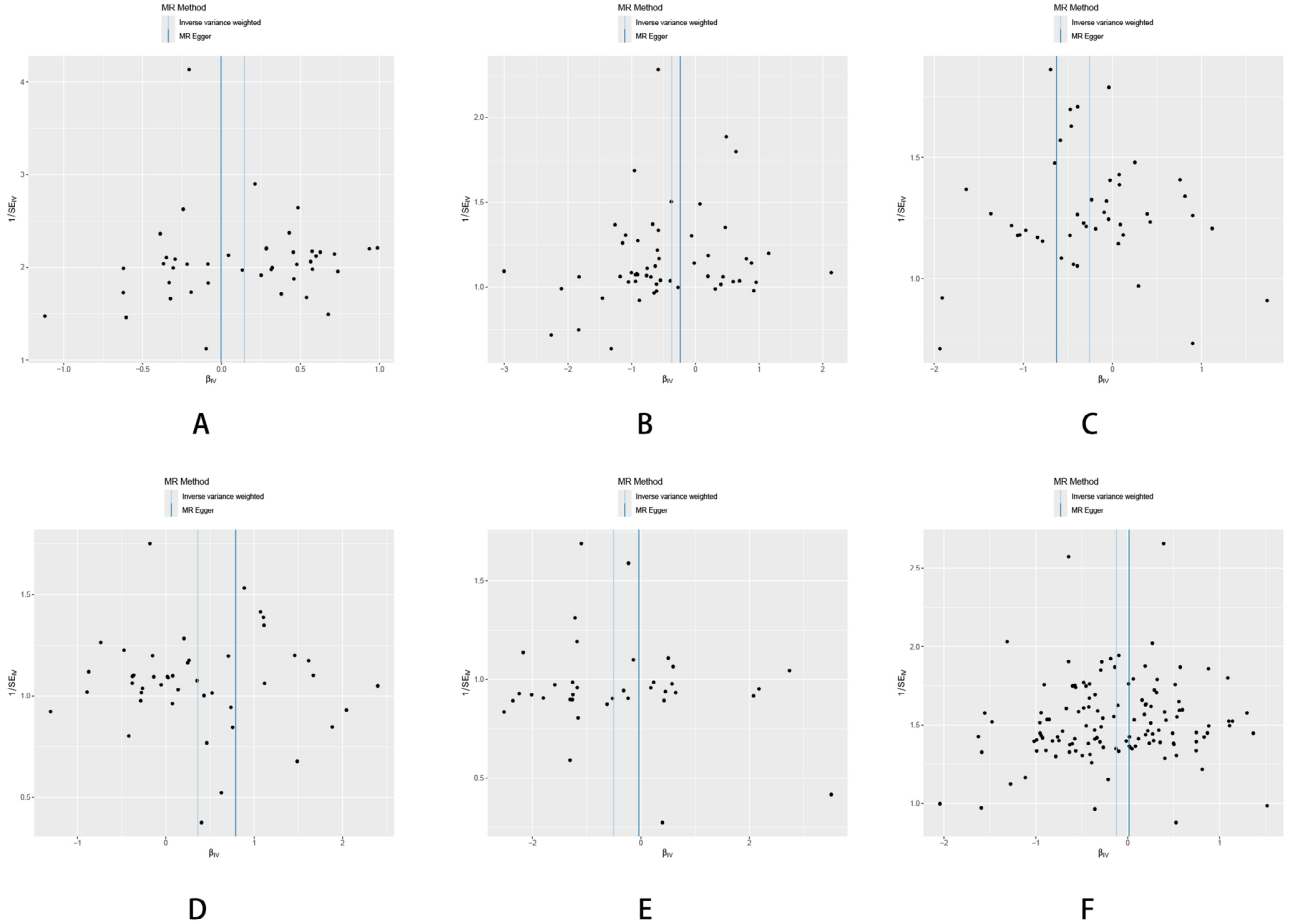
Funnel plot for the overall heterogeneity between: F‐caffeinated/sweet liking (A), crisps liking (B), curry liking (C), jam liking (D), oranges liking (E), and F‐strong flavor liking (F).

**FIGURE 7:**
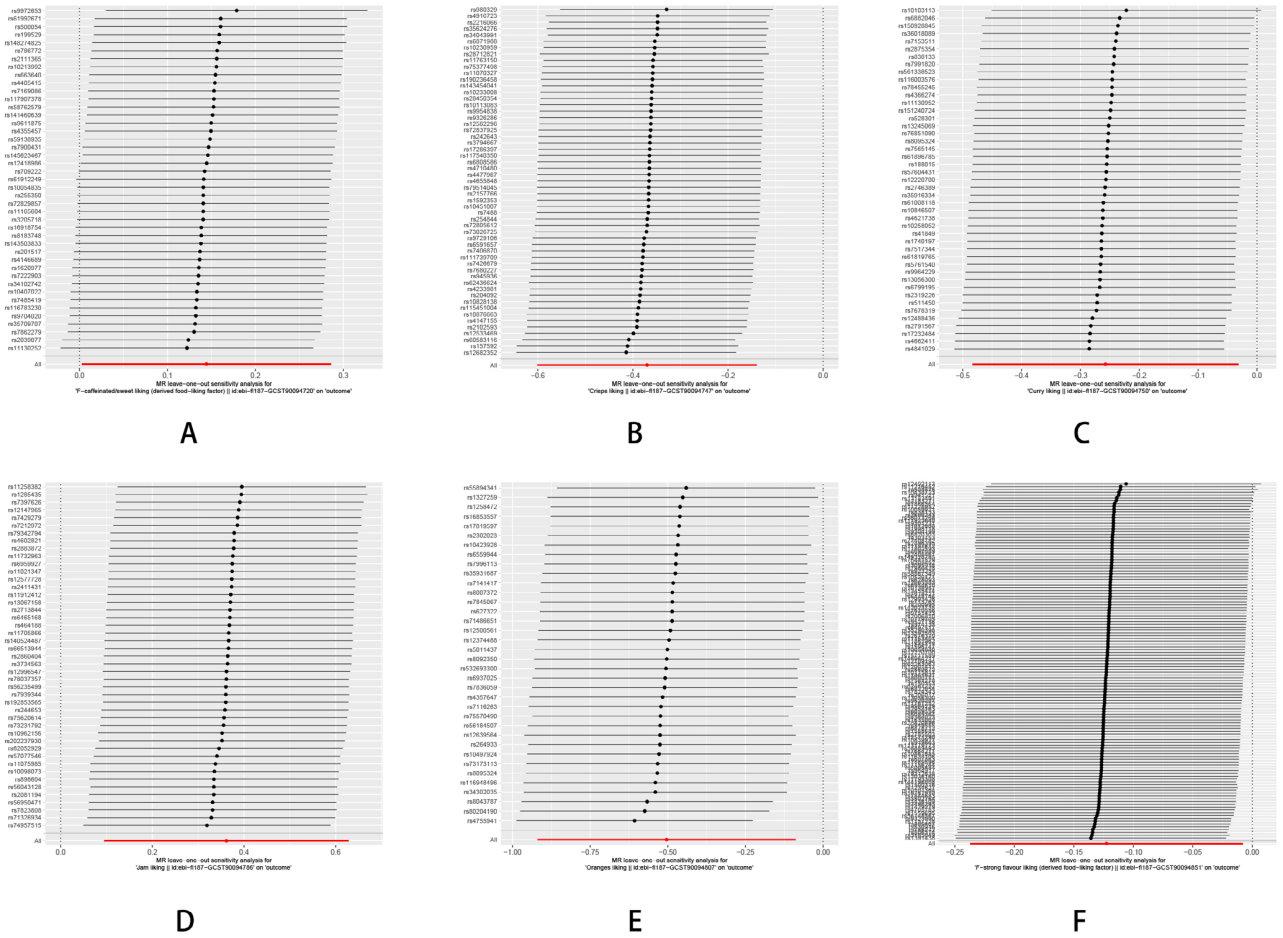
Leave one out analysis of the MR results between specific dietary habits and HTS: (A) F‐caffeinated/sweet liking; (B) crisps liking; (C) curry liking; (D) jam liking; (E) oranges liking; (F) F‐strong flavor liking.

Despite this, the analyses for F‐caffeinated/sweet liking, crisps liking, curry liking, jam liking, and strong flavor liking revealed no significant evidence of heterogeneity or pleiotropy, indicating that the causal estimates for these exposures are robust and reliable. MR‐Egger regression intercepts further supported the validity of the genetic instruments, as no significant evidence of directional pleiotropy was detected. These results collectively indicate that, while heterogeneity was observed for oranges liking, the overall findings for the remaining exposures are consistent and provide reliable causal evidence (Figures 6 and 7 and Table S3A).

The MR Steiger test confirmed the correct directionality for all analyses, indicating that the SNPs were more strongly associated with the exposure than the outcome (Table S5).

### 5.2 Effect of Gut Microbiota on HTS

The MR analysis examining the causal relationship between gut microbiota composition and HTS identified several significant associations using the inverse‐variance weighted (IVW) method. Among the taxa analyzed, the genus *Eubacterium coprostanoligenes* group showed a statistically significant protective effect on HTS, with an odds ratio (OR) of 0.54 (95% CI: 0.33–0.87, *p* = 0.012), indicating that a greater abundance of this genus reduces the risk of HTS. Similarly, the genus Collinsella demonstrated a negative association with HTS, with an OR of 0.48 (95% CI: 0.24–0.95, *p* = 0.035), suggesting a potential protective role.

**FIGURE 8:**
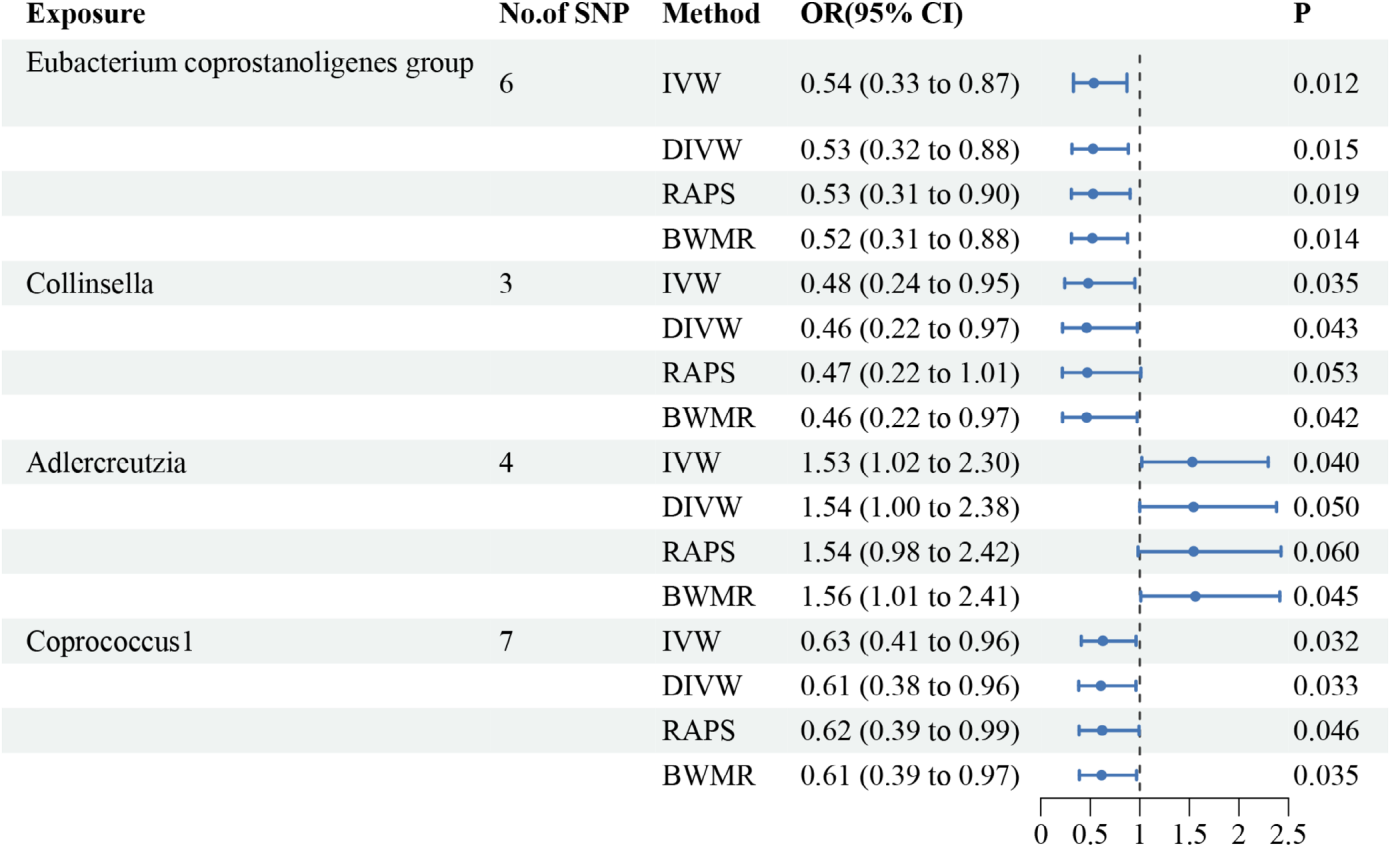
Forest plot summarizing MR estimates for the associations between gut microbiota taxa and genetically predicted HTS.

**FIGURE 9:**
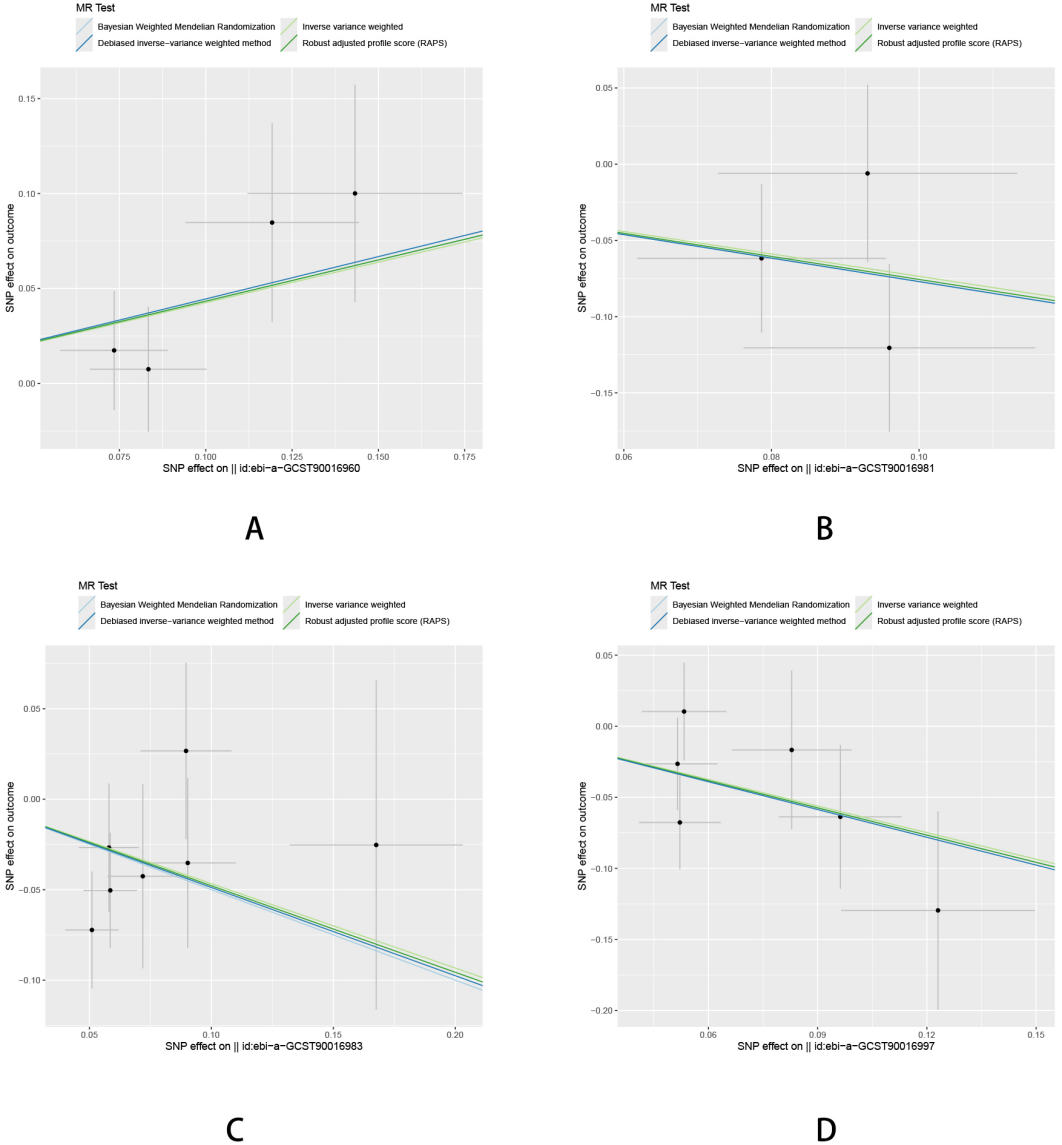
The effect of gut microbiota on HTS. (A) Genus Adlercreutzia; (B) Collinsella; (C) Coprococcus1; (D) *Eubacterium coprostanoligenes* group.

In contrast, the genus Adlercreutzia showed a significant positive association with HTS, with an OR of 1.53 (95% CI: 1.02–2.30, *p* = 0.040), indicating an increased risk of HTS with higher abundance of this genus. A similar result was observed for the genus Coprococcus1, which exhibited a negative association with HTS, with an OR of 0.63 (95% CI: 0.41–0.96, *p* = 0.032), suggesting a protective effect (Figures 8 and 9 and Table S2).

**FIGURE 10:**
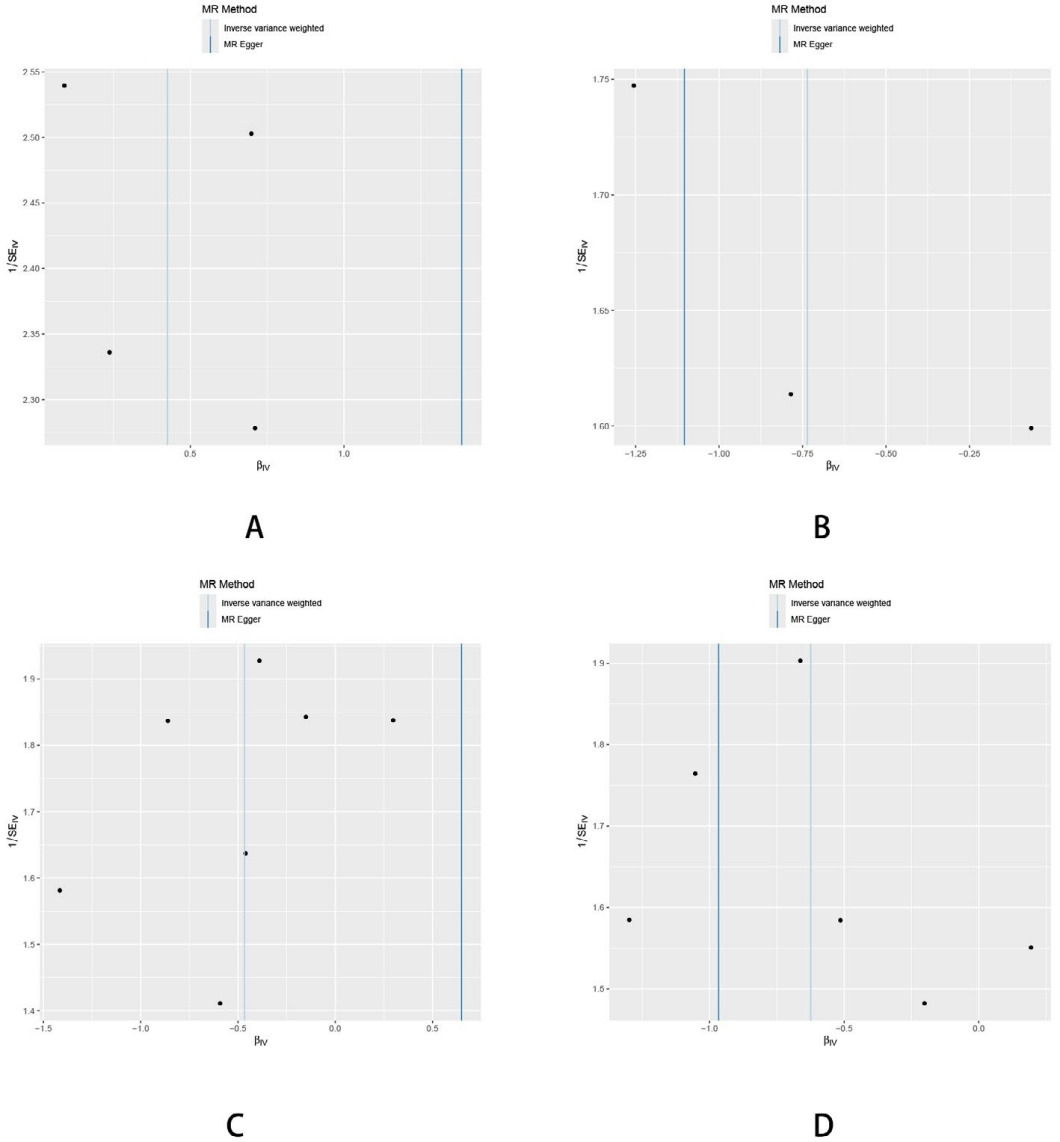
The funnel plot illustrates the overall heterogeneity between gut microbiota and HTS. (A) Genus Adlercreutzia; (B) Collinsella; (C) Coprococcus1; (D) *Eubacterium coprostanoligenes* group.

**FIGURE 11:**
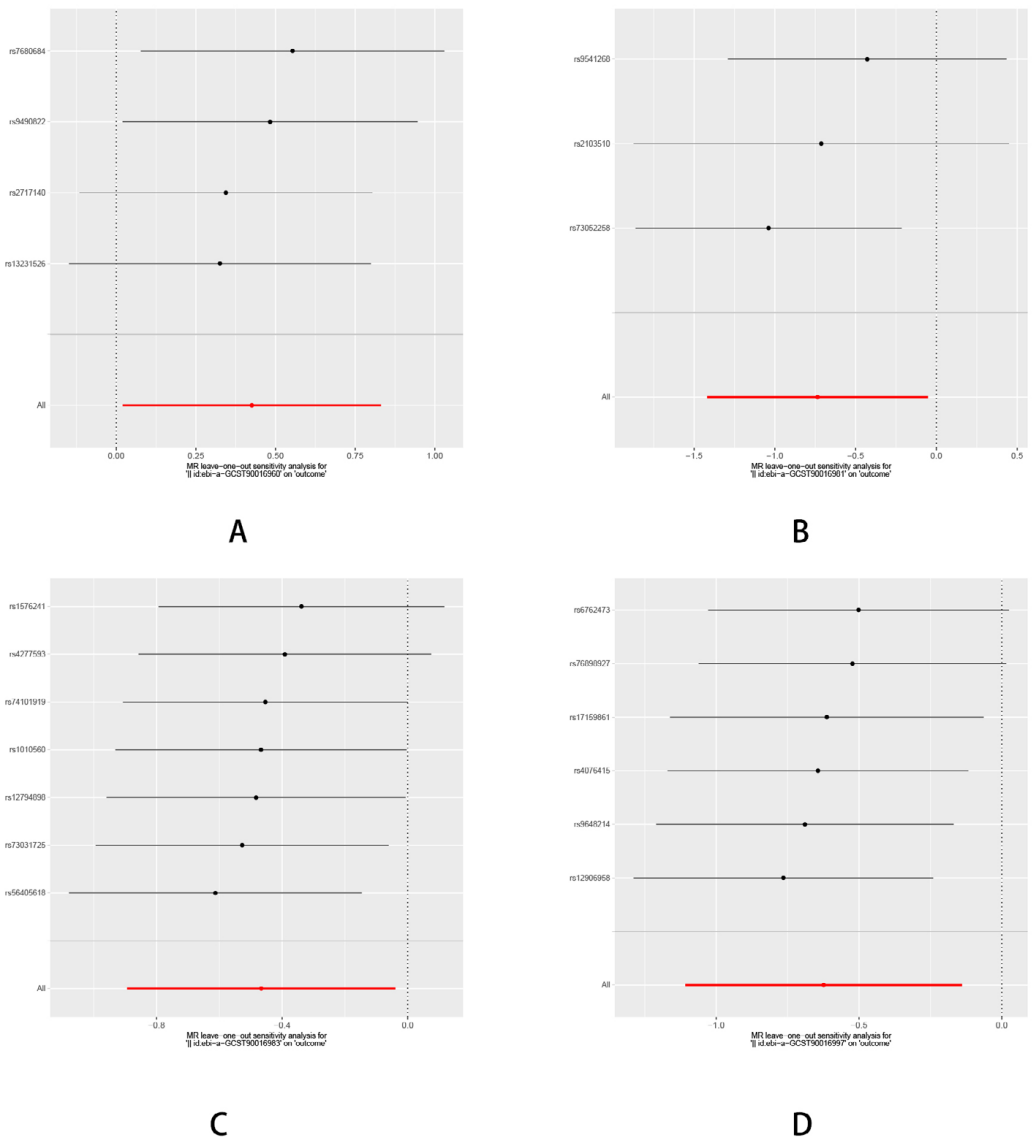
The leave one out analysis of the MR results between gut microbiota and HTS. (A) Genus Adlercreutzia; (B) Collinsella; (C) Coprococcus1; (D) *Eubacterium coprostanoligenes* group.

Sensitivity analyses were performed to confirm the robustness of these findings, including tests for horizontal pleiotropy using MR‐Egger regression and assessments of heterogeneity with Cochran's Q statistic. For all significant results, no evidence of pleiotropy was detected, and the estimates were consistent across genetic variants, as verified through leave‐one‐out analyses. These findings underscore the potential role of gut microbiota composition in influencing the risk of HTS, with distinct genera showing either protective or detrimental effects (Figures 10 and 11 and Table S3B).

The MR Steiger test confirmed the correct directionality for all analyses, indicating that the SNPs were more strongly associated with the exposure than the outcome (Table S5).

### 5.3 Effect of Food Liking on Gut Microbiota

In a follow‐up MR analysis exploring whether the six identified dietary habits influence HTS risk through alterations in gut microbiota composition, only jam liking demonstrated a statistically significant association with the Coprococcus1 genus. Specifically, jam liking was negatively associated with the abundance of Coprococcus1, suggesting a potential protective effect against HTS development.

For the other dietary habits and gut microbiota taxa analyzed, no significant associations were observed (all *p*‐values > 0.05). These results indicate that, among the dietary exposures examined, only jam liking showed evidence of influencing gut microbiota composition in a manner potentially relevant to HTS development (Table S4).

### 5.4 Mediated Effect of Gut Microbiota

The mediation analysis assessed whether the Coprococcus1 genus mediates the relationship between jam liking and HTS. The total effect was statistically significant, indicating a positive association between jam liking and HTS. The direct effect also reached significance, demonstrating that this association largely operates independently of gut microbiota mediation. However, the intermediary effect was not statistically significant (*p* = 0.145), with only 14.3% of the total effect estimated to be mediated through Coprococcus1. These findings suggest that while dietary habits significantly influence HTS, the mediating role of gut microbiota was not robustly supported in this analysis (Table 1).

**TABLE 1:** Dietary habits significantly influence HTS; the mediating role of gut microbiota was not robustly supported in this analysis.

Effect type	Beta	OR	95% CI	*p*‐value	Mediated proportion (%)
Total Effect (TE)	0.362	1.44	1.10–1.87	0.0079	—
Direct Effect (DE)	0.31	1.36	1.03–1.80	0.0283	—
Intermediary Effect (IE)	0.052	1.05	0.98–1.13	0.145	14.3 (−6.1 to 34.7)

### 5.5 Co‐Localization Analysis

**FIGURE 12:**
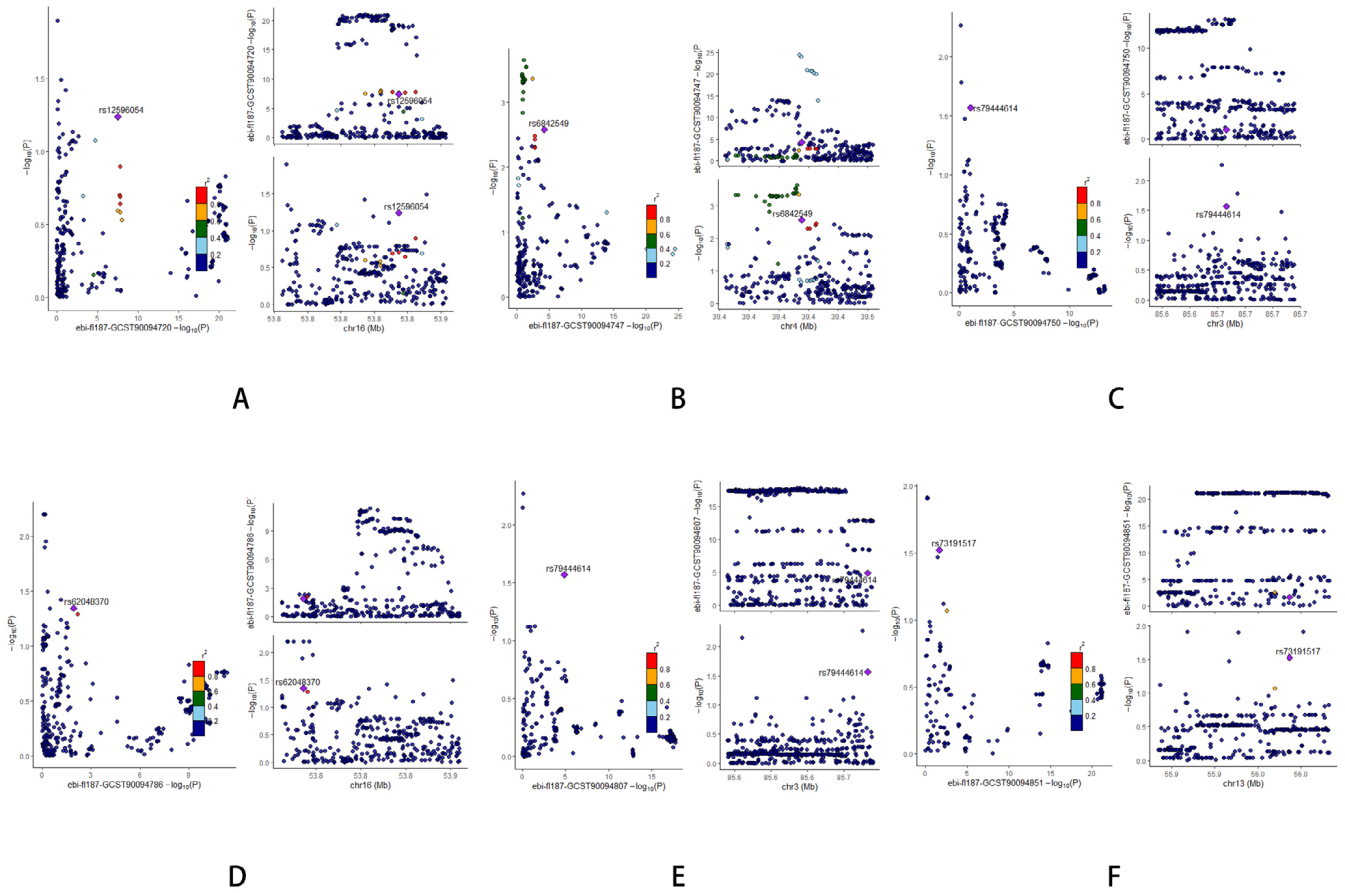
Colocalization analysis results for the association between specific dietary habits and HTS: (A) F‐caffeinated/sweet liking; (B) crisps liking; (C) curry liking; (D) jam liking; (E) oranges liking; (F) F‐strong flavor liking.

**FIGURE 13:**
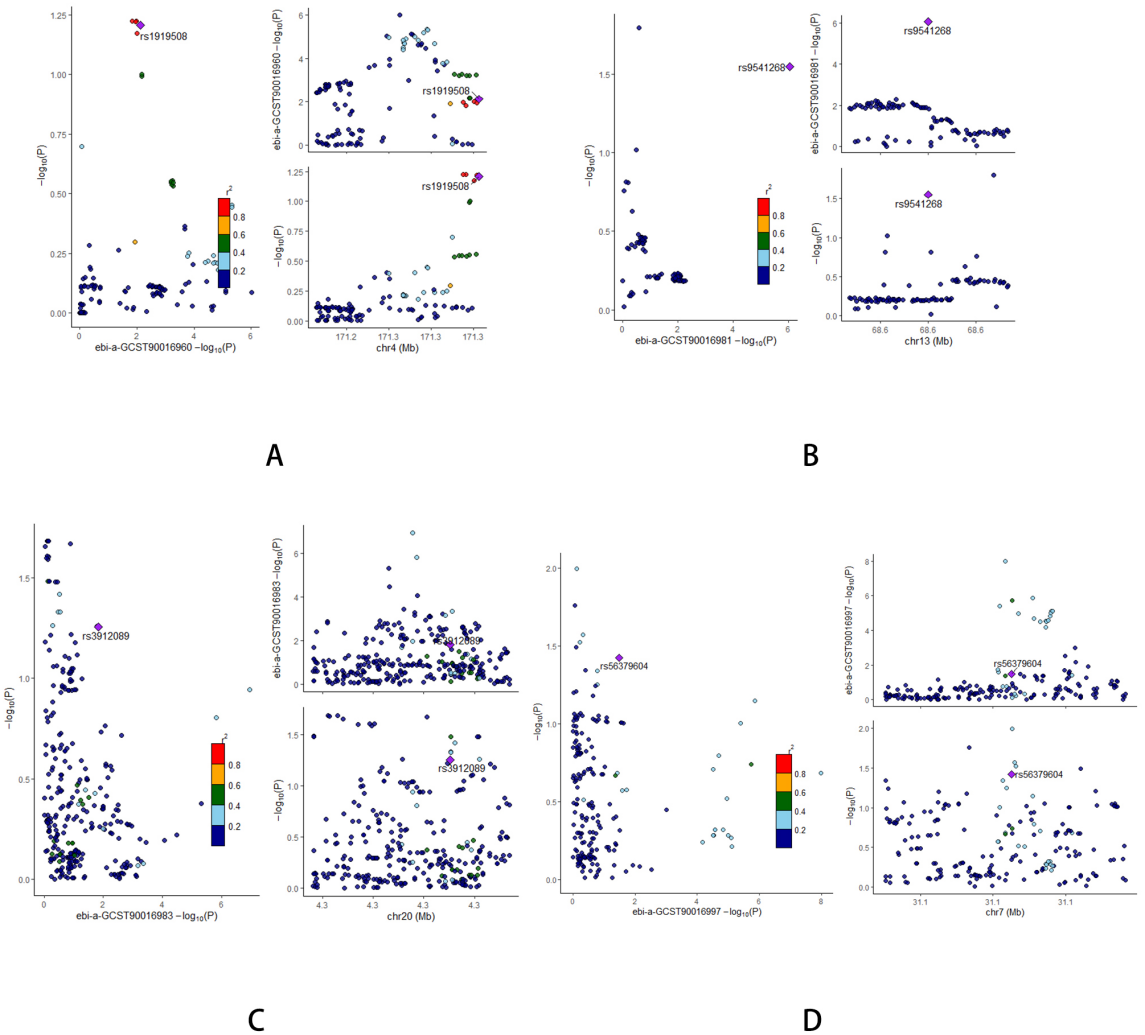
Colocalization analysis results for the association between specific gut microbiota and HTS: (A) Genus Adlercreutzia; (B) Collinsella; (C) Coprococcus1; (D) *Eubacterium coprostanoligenes* .

The co‐localization analysis was performed to assess whether dietary habits and gut microbiota abundance share common causal genetic variants with HTS. The posterior probability for hypothesis 4 was used as the primary indicator, where values below 0.75 suggest that the traits are unlikely to share a common genetic variant. For dietary habits, jam liking showed the highest PP.H4 value at 3.46E‐02, followed by crisps liking and strong flavor liking, with values of 2.91E‐02 and 2.79E‐02, respectively. Other dietary traits, including curry liking and oranges liking, exhibited lower PP.H4 values of 1.70E‐02 and 1.77E‐02, indicating minimal evidence for shared genetic loci with HTS. Similarly, for gut microbiota, the Eubacterium coprostanoli genes group and Coprococcus1 genus displayed the highest PP.H4 values at 4.84E‐02 and 4.38E‐02, respectively. However, these values remain below the threshold, suggesting that these microbial taxa do not share significant causal genetic variants with HTS (Figures 12 and 13 and Table S6). These findings indicate that neither the dietary habits nor gut microbiota analyzed are likely to share common genetic variants with HTS, implying that their associations with HTS may be driven by distinct genetic factors.

## 6 Discussion

This study utilized a Mendelian randomization framework to explore the causal links between dietary preferences, gut microbiota composition, and the risk of HTS. Drawing from genome‐wide association study summary statistics within MR design, we identified several dietary traits—including preferences for caffeinated and sweet items, jam, crisps, curry, oranges, and strong flavors—as significantly associated with HTS susceptibility (Figures 4 and 8). In parallel, specific gut microbial taxa such as *Eubacterium coprostanoligenes* , Coprococcus1, Collinsella, and Adlercreutzia were found to exert potential causal effects on HTS development. These microbial features demonstrated both protective and deleterious roles, likely through diverse immunological, metabolic, and fibrotic pathways, reflecting the taxon‐specific complexity of the gut ecosystem's contribution to tissue remodeling.

Although mediation analysis suggested that gut microbiota might account for approximately 14.3% of the dietary effect on HTS, the observed mediation effects did not achieve statistical significance (Table 1). This result implies that the influences of diet and microbiota on HTS may unfold through biologically independent trajectories. Co‐localization analysis further supported this interpretation by demonstrating that the posterior probabilities of shared causal variants between microbial traits and HTS remained below accepted thresholds. Together, these findings provide compelling evidence that dietary exposures and microbial composition contribute to HTS risk through distinct physiological mechanisms. They also underscore that the gut–skin connection reflects an integrated network shaped by exogenous nutrients and endogenous microbial activity, each modulating inflammatory tone and regenerative outcomes through separate yet converging pathways.

Dietary behaviors are widely recognized as key regulators of cutaneous wound healing, influencing inflammatory resolution, oxidative balance, and collagen matrix turnover (Zhao et al. 2018). In the present study, six dietary preferences were found to have putative causal roles in HTS development, reinforcing the importance of nutrition in fibrotic skin processes. Preferences for caffeinated and sweet foods were positively associated with increased HTS risk. Mechanistically, high sugar consumption facilitates the accumulation of advanced glycation end‐products (AGEs), which compromise collagen integrity and promote fibroblast dysfunction by stiffening the extracellular matrix and sustaining chronic inflammation (Erim and Binici 2024; Mony et al. 2023). A similar risk profile was observed with jam consumption, likely driven by its high refined sugar content and its association with pro‐inflammatory dietary patterns characterized by low intake of antioxidant‐rich plant foods (Dunnill et al. 2017; Xuan et al. 2014).

Conversely, several dietary features appeared to mitigate HTS risk. Despite their general categorization as unhealthy, crisps showed a protective association, possibly owing to the inclusion of polyunsaturated fatty acids such as linoleic and alpha‐linolenic acids in some formulations. These lipids have demonstrated immunomodulatory effects, attenuating cytokine production and promoting keratinocyte migration and balanced fibroblast activity during wound repair (Peng et al. 2018; Ishak et al. 2019). Curry consumption was similarly protective, likely due to the presence of polyphenols such as curcumin, gingerol, and cumin aldehyde. These compounds have been shown to suppress TGF‐β signaling, modulate macrophage polarization, and restore redox equilibrium in fibrotic models (Wang et al. 2024; Ashrafizadeh et al. 2020).

The beneficial effect associated with orange preference is plausibly linked not only to its vitamin C content, a known cofactor in collagen stabilization, but also to flavonoids like hesperidin and narirutin that inhibit matrix metalloproteinase activity and support re‐epithelialization (Thevi et al. 2024; Mohammed et al. 2016). A liking for strong flavors may also reflect frequent intake of spices such as oregano, rosemary, or garlic, which possess bioactive components capable of modulating immune responses, reducing microbial burden, and regulating fibroblast contraction through nitric oxide and NF‐κB pathways (Süntar et al. 2011; Yu, Ma et al. 2023). These combined dietary patterns suggest that plant‐derived phytochemicals may reshape the cutaneous microenvironment in a manner favorable to scar attenuation (Azeredo et al. 2024).

Despite establishing causal links between specific dietary traits and microbial taxa with HTS risk, subsequent mediation MR analysis did not identify statistically significant intermediary effects of the gut microbiota. Although an estimated 14.3% of the total dietary influence might be mediated through microbiota, this proportion lacked statistical robustness. The implication is that diet and microbial taxa likely operate along distinct mechanistic routes. Dietary constituents may act directly via systemic metabolic pathways or hormonal regulation, whereas gut microbes might affect HTS risk by modulating host immunity, producing signaling metabolites, and influencing intestinal barrier integrity.

Closer examination of microbial contributions revealed notable taxon‐specific patterns. For instance, *Eubacterium coprostanoligenes* and Coprococcus1, known butyrate producers, have been associated with anti‐fibrotic effects by suppressing proinflammatory cytokines, enhancing regulatory T cell development, and inhibiting histone deacetylation, thus modulating fibroblast gene expression and collagen synthesis (Machado et al. 2021). Collinsella, linked to bile acid metabolism, may stabilize epithelial function and contribute to immunological tolerance, thereby limiting excessive tissue remodeling (Overby and Ferguson 2021; Freier et al. 1994; Midgley et al. 2013; Oñate et al. 2023). In contrast, Adlercreutzia, through its metabolism of dietary phytoestrogens, may produce pro‐inflammatory metabolites that exacerbate wound healing responses and promote fibrosis. These contrasting effects emphasize the need for microbiota analysis at the genus or species level, as different taxa may exert divergent impacts on fibrotic progression (Gargari et al. 2024; Hirayama et al. 2021).

To investigate whether genetic overlap underpinned these observed associations, we performed co‐localization analyses for each significant exposure–outcome pair. Across all tested combinations, the posterior probabilities for shared causal variants did not reach the conventional threshold, suggesting that the associations are unlikely to stem from pleiotropic genetic effects. Instead, the results point to independent genetic architectures governing dietary behavior, microbial composition, and HTS susceptibility. Such a pattern supports the idea that these traits are causally linked through functionally related but genomically distinct pathways.

Taken together, these results underscore the complexity of the interactions among host diet, microbiota, and scarring. They also suggest that preventive strategies targeting either diet or microbial composition may operate through complementary and independent mechanisms, offering the potential for synergistic therapeutic interventions.

Several important considerations should be taken into account when interpreting the findings of this study. The analyses were based on summary‐level genome‐wide association data, which did not allow for individual‐level adjustment of potential confounders, such as age, body mass index, comorbidities, or probiotic and antibiotic usage. Although Mendelian randomization is designed to reduce confounding, residual bias related to these unmeasured variables cannot be entirely ruled out.

The datasets used in this study were derived from large public biobanks, including the MiBioGen consortium for gut microbiota and the FinnGen biobank for hypertrophic scarring. While these resources offer considerable statistical power and technical consistency, the predominance of participants with European ancestry may limit the extrapolation of findings to more diverse populations. Moreover, the microbial data were restricted to bacterial taxa, leaving other microbial domains, particularly the gut mycobiome and virome, unaddressed in the current causal framework.

Another limitation lies in the assessment of dietary exposures, which was based on self‐reported food preference data. Although these data have demonstrated genetic validity in previous analyses, self‐reported preferences do not necessarily reflect actual intake, dietary frequency, or nutrient bioavailability, all of which are important in shaping metabolic and immune pathways involved in fibrosis.

The null findings in the mediation and co‐localization analyses may reflect limited statistical power or intrinsic methodological constraints, particularly in detecting modest effects or resolving shared causal variants across complex traits. These aspects, while not diminishing the validity of the primary findings, do highlight the challenges of disentangling causal hierarchies in multifactorial traits such as hypertrophic scarring.

Future research should move toward resolving these uncertainties by employing high‐resolution, individual‐level data that incorporate detailed clinical, dietary, and lifestyle profiles. Expanding the representation of non‐European populations will be essential for improving the generalizability of findings, and the inclusion of underexplored microbial groups such as fungi and viruses may help complete the picture of host–microbe interactions in scar pathophysiology.

In addition, longitudinal studies tracking changes in diet, microbiota, and wound healing outcomes over time could provide stronger evidence for causal directionality. Multi‐omics approaches integrating metagenomic, transcriptomic, and metabolomic data are likely to be particularly informative in identifying the biological intermediates that link external exposures to fibrotic skin outcomes. Exploring how microbial‐derived metabolites interact with dietary components to modulate fibroblast activation, collagen remodeling, and inflammatory signaling could further clarify the mechanistic basis of these associations.

From a translational perspective, the development of targeted interventions, including dietary modifications or microbiota‐directed therapies, holds promise for mitigating the risk of hypertrophic scarring. Interventional studies designed to test the efficacy of antioxidant‐rich diets, low‐glycemic regimens, or the use of probiotic formulations may provide practical strategies for clinical prevention. Building on the insights from this genetic analysis, such work has the potential to bridge population‐level observations with individualized therapeutic approaches.

## 7 Conclusion

This study identified significant causal links between dietary habits, gut microbiota composition, and hypertrophic scar (HTS) risk, revealing both protective and risk‐enhancing effects of specific food preferences and microbial taxa. These findings underscore the role of systemic metabolic and inflammatory pathways in scar development and support the potential utility of microbiota‐ and diet‐based preventive strategies. As current analyses were limited to European populations and summary‐level data, future research should aim to validate these findings in multi‐ethnic cohorts and explore mechanistic pathways using integrated multi‐omics and longitudinal designs.

## Author Contributions

**Qiong Liu:** funding acquisition (lead), investigation (equal), methodology (equal), resources (equal), software (equal), visualization (equal), writing – original draft (equal). **Xiaofang Liu:** conceptualization (equal), investigation (equal), methodology (equal), resources (equal), software (equal), validation (equal). **Mengge Gao:** conceptualization (equal), data curation (equal), formal analysis (equal), methodology (equal), resources (equal), software (equal), supervision (equal), validation (equal), visualization (equal), writing – original draft (equal). **Bo Yang:** data curation (equal), formal analysis (equal), funding acquisition (equal), investigation (equal), methodology (equal), software (equal), validation (equal), visualization (equal), writing – original draft (equal). **Miaoqing Luo:** conceptualization (equal), software (equal), writing – original draft (equal). **Biying Yang:** formal analysis (equal), methodology (equal). **Guojun Liang:** project administration (lead), supervision (lead), writing – original draft (equal), writing – review and editing (lead).

## Ethics Statement

The authors have nothing to report.

## Conflicts of Interest

The authors declare no conflicts of interest.

## Supporting information


Table S1–S6.

## Data Availability

All data generated or analyzed during this study are included in this published article and its Supporting Information files.
